# Higher plasma prorenin concentration plays a role in the development of coronary artery disease

**DOI:** 10.1186/s40364-015-0044-1

**Published:** 2015-07-11

**Authors:** Gakuro Yoshida, Masanori Kawasaki, Ichijiro Murata, Yuka Hayakawa, Takuma Aoyama, Nagisa Miyazaki, Yoshihisa Yamada, Kazuhiko Nishigaki, Yoshie Arai, Fumiaki Suzuki, Shinya Minatoguchi

**Affiliations:** Department of Nephrology, Gifu University Graduate School of Medicine, Gifu, Japan; Department of Cardiology, Gifu University Graduate School of Medicine, Yanagido 1-1, Gifu, 501-1194 Japan; Faculty of Applied Biological Sciences, Gifu University, Gifu, Japan

**Keywords:** Prorenin, Renin, Coronary artery disease

## Abstract

**Background:**

Prorenin and renin are both involved in atherosclerosis. However, the role of plasma prorenin and renin in the development and progression of coronary artery disease (CAD) is still not clear. Thus, we aimed to examine the relationships among plasma prorenin concentration, CAD and clinical parameters.

**Methods:**

We measured plasma prorenin and renin concentrations and other parameters in 85 patients who underwent coronary angiography. Patients were divided into a CAD group (≥75 % stenosis in one or more coronary arteries) and a non-CAD group.

**Results:**

There was a weak correlation between prorenin and plasma renin concentration (r =0.35, p =0.001), and plasma renin activity (r =0.34, p =0.001). There was no significant difference in the plasma prorenin concentration between the CAD group and non-CAD group. However, patients with a high plasma prorenin concentration frequently suffered CAD. Receiver-operating-characteristic curve analysis showed that the optimal cutoff value of plasma prorenin concentration to detect CAD was 1,100 pg/ml, with a positive predictive value of 94 % and a negative predictive value of 36 %.

**Conclusion:**

The plasma prorenin concentration increases with increases in plasma renin concentration. Higher plasma prorenin concentration (>1,100 pg/ml) plays a role in the development of CAD.

## Introduction

The renin-angiotensin system is a key regulator of the cardiovascular system. The principal effector hormone of the classic renin-angiotensin system is angiotensin II. The conversion of angiotensinogen to angiotensin I by renin is the rate limiting step in the synthesis of renin-angiotensin II. Chronic activation of renin-angiotensin system is a major contributing factor to the pathogenesis and progression of cardiovascular disease. In fact, higher plasma renin activity (PRA) was reported to be associated with an increased risk of myocardial infarction in the patients with hypertension [1]. Furthermore, another recent study demonstrated that high PRA was associated with an increased cardiac morbidity and mortality in patients with coronary artery disease (CAD) [2].

For many years, prorenin was considered to be an inactive precursor of renin. However, since the discovery of (pro)renin receptors, prorenin is now regarded as an important regulator of the renin-angiotensin system. Both prorenin and renin bind to (pro)renin receptors, and stimulation of (pro)renin receptors has been reported to be involved in the development of diabetic nephropathy [3] cardiac injury [4] and vascular damage [5]. Prorenin is released constitutively from the kidney, and its blood plasma levels are approximately 10-fold higher than those of renin. Chronic stimulation of the renin-angiotensin system usually increases renal prorenin-renin conversion, thereby decreasing the relative amount of prorenin in the circulation [6]. Furthermore, plasma prorenin concentration is markedly increased in diabetes mellitus patients with complications [7]. However, the plasma prorenin concentration and its pathophysiological role in patients with CAD are not clear. Therefore, in the present study, we aimed to examine the relationships among plasma prorenin concentration, CAD and clinical parameters.

## Methods

### Study patients

We initially enrolled 100 consecutive patients with chest pain and/or chest discomfort who underwent elective coronary angiography for suspicion of CAD. We excluded subjects with unstable angina or myocardial infarction within the previous three months and severe heart failure (NYHA class IV and/or a left ventricular ejection fraction ≤ 30 %). The final study population consisted of 85 patients who underwent coronary angiography. Patients were diagnosed with CAD when one or more of their coronary arteries had a stenosis ≥75 %. According to the angiographical diagnosis, patients were divided into two groups (CAD group or non-CAD group). Risk factors for CAD were evaluated in enrolled patients, including hypertension (HTN) (medication-dependent or systolic BP ≥140 and/or diastolic BP ≥90 mmHg), type 2 diabetes mellitus (DM) (medication-dependent or hemoglobin (Hb) A1c ≥ 6.5 %) and dyslipidemia (medication-dependent, LDL cholesterol ≥140 mg/dl and/or HDL cholesterol <40 mg/dl). The protocol was approved by the ethics committee of Gifu University Graduate School of Medicine. All of the patients gave informed consent before the start of study. The investigation conformed with the principles outlined in the Declaration of Helsinki (Br Med J 1964; ii:177).

### Hemodynamic parameters

Blood pressure and pulse rate were measured. Left ventricular ejection fraction (LVEF) left ventricular end-diastolic dimension (LVEDd) and left ventricular end-systolic dimension (LVESd) were obtained by echocardiography. Echocardiographic measurements were used to calculate LVEF based on a disk summation method using the apical 2- and 4-chamber views.

### Measurements of plasma prorenin and renin concentration

Blood samples were obtained from the antecubital vein in the morning while the patients were in a fasting state on the day of cardiac catheterization. The samples were collected into sterile tubes, immediately placed on ice, centrifuged at 3,000 x g for 10 min at 4 °C, and rapidly frozen and stored at −80 °C until analysis. Plasma prorenin concentrations were measured using an ELISA kit (LINCO Research Inc., St. Charles, MO, USA). Plasma renin concentrations were also measured using an ELISA kit (LINCO Research Inc., St. Charles, MO, USA). Plasma brain natriuretic peptide (BNP) levels were measured by immunoradiometric assay (Shionoria BNP RIA kit; Shionogi, Osaka, Japan). We also measured hemoglobin A1c (HbA1c), total cholesterol, LDL-cholesterol, HDL-cholesterol and estimated glomerular filtration rate (eGFR).

### Statistical analysis

The data are shown as the mean ± one standard deviation. Categorical data were summarized as percentages and compared with a chi-square test or Fisher’s exact test. The normality of data distributions was tested using the Kolmogorov-Smirnov test. The significance of the differences between groups for variables that were normally distributed and had similar variances was determined by an unpaired Student’s *t* test. Otherwise, a Mann–Whitney U test was used to compare the differences between groups. Linear regression analysis was performed to examine the correlations between plasma prorenin concentration and other parameters. A p value <0.05 was considered significant. All statistical analyses were performed using Stat View version 5.0 (SAS Institution Inc, Cary, NC, USA).

## Results

### Patient’s characteristics

Patient characteristics are shown in Table 1. The patients were taking oral angiotensin-converting enzyme inhibitors (ACEIs) or angiotensin II receptor blockers (ARBs) (n =24), calcium channel blockers (n =30), beta-blockers (n =12), or diuretics (n =9). There were no significant differences between the two groups in history of hypertension, current smoking or concomitant medication use. However, history of DM, history of dyslipidemia and age were significantly greater in the CAD group than in the non-CAD group.

**Table 1:** Patient clinical characteristics and echocardiographic parameters

	Non-CAD group	CAD group	p-value
	(n = 26)	(n = 59)	
Clinical characteristics			
Men (%)	20 (76.9)	39 (66.1)	0.27
Age, years	62 ± 15	69 ± 9	0.007
Body mass index	22.7 ± 3.1	23.8 ± 3.0	0.12
Hypertension, n (%)	12 (46.1)	36 (61.0)	0.15
Diabetes mellitus, n (%)	5 (19.2)	28 (47.5)	0.010
Dyslipidemia, n (%)	5 (19.2)	32 (56.1)	0.002
Systolic blood pressure (mmHg)	125 ± 18	130 ± 18	0.21
Diastolic blood pressure (mmHg)	75 ± 11	75 ± 12	0.85
Echocardiographic parameters			
Left ventricular ejection fraction (%)	70 ± 6	62 ± 11	0.001
LVEDd (mm)	45 ± 7	47 ± 5	0.15
LVSWth (mm)	9.2 ± 1.7	9.5 ± 1.3	0.41
LVPWth (mm)	9.6 ± 1.5	9.7 ± 1.5	0.72
Medications			
ARBs or ACEIs, n (%)	8 (30.8)	16 (27.1)	0.81
Beta-blockers, n (%)	3 (11.5)	9 (15.2)	0.61
Calcium channel blockers, n (%)	7 (26.9)	23 (39.0)	0.23
Diuretics, n (%)	2 (7.7)	7 (11.9)	0.71

LVEDd: Left ventricular end-diastolic dimension, LVSWth: left ventricular septal wall thickness, LVPWth: left ventricular posterior wall thickness, ACEIs: angiotensin-converting enzyme inhibitors, ARBs: Angiotensin II receptor blockers

### Hemodynamic parameters

As shown in Table 2, there were no significant differences in heart rate, systolic blood pressure, diastolic blood pressure or LVEDd between the CAD group and the non-CAD group. The LVEF was significantly greater in the non-CAD group than in the CAD group.

**Table 2:** Patient laboratory characteristics

	Non-CAD group	CAD group	p-value
	(n = 26)	(n = 59)	
Prorenin (pg/ml)	490 (321–786)	522 (297–1097)	0.70
Plasma renin concentration (pg/ml)	5.8 (3.3 - 9.8)	6.3 (4.4 - 12.0)	0.84
Plasma renin activity (ng/ml/hr)	1.0 (0.5 - 2.1)	1.1 (0.6 - 2.4)	0.83
Aldosterone (pg/ml)	85 (65–130)	74 (59–98)	0.40
Brain natriuretic peptide (ng/ml)	20 (14–55)	32 (14–62)	0.38
Cystatin C (mg/dl)	1.09 ± 0.87	1.09 ± 0.38	0.97
Creatinine (mg/ml)	1.01 ± 1.48	0.85 ± 0.27	0.42
Estimated GFR (ml/min/1.73 m^2^)	77.1 ± 28.3	69.6 ± 19.2	0.16
Blood urea nitrogen (mg/dl)	18.0 ± 20.1	15.9 ± 4.1	0.45
Hemoglobin A1c (%)	5.7 ± 0.7	6.1 ± 1.1	0.16
Total cholesterol (mg/dl)	191 ± 43	187 ± 33	0.75
LDL cholesterol (mg/dl)	115 ± 39	112 ± 31	0.71
HDL cholesterol (mg/dl)	51 ± 13	48 ± 14	0.39
Triglycerides (mg/dl)	114 ± 56	139 ± 72	0.11
C-reactive protein (mg/dl)	0.24 ± 0.52	0.18 ± 0.21	0.52
Total protein (g/dl)	6.68 ± 0.52	6.72 ± 0.43	0.68
Albumin (g/dl)	3.99 ± 0.32	4.13 ± 0.30	0.054
Sodium (mEq/l)	141.1 ± 2.1	140.3 ± 2.3	0.57
Potassium (mEq/l)	4.10 ± 0.41	4.05 ± 0.35	0.53
Cl (mEq/l)	105.3 ± 3.2	105.4 ± 2.7	0.92

Numerical data are expressed as the mean ± one standard deviation. Non-parametric data are expressed as the median (interquartile range). GFR: glomerular filtration rate

### Plasma prorenin concentration and plasma renin concentration

**Fig. 1:**
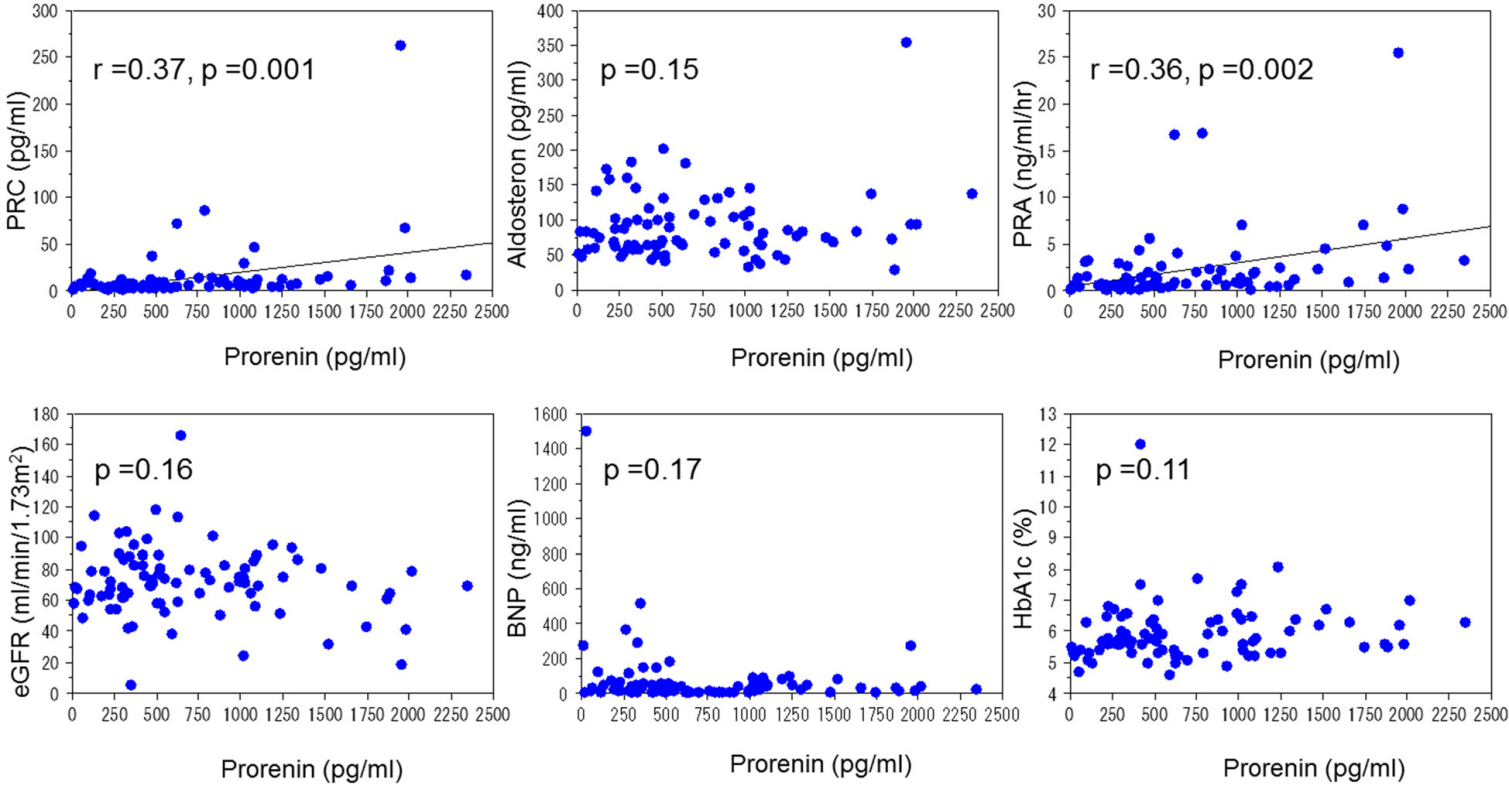
Relationship between plasma prorenin concentration and laboratory parameters. PRC: plasma renin concentration, PRA: plasma renin activity, HbA1c: hemoglobin A1c, BNP: brain natriuretic peptide, eGFR: estimated glomerular filtration rate

There was a weak correlation between the plasma prorenin concentration and plasma renin concentration (r =0.35, p =0.001), and plasma renin activity (r =0.34, p =0.001) (Fig. 1). However, there was no correlation between the plasma prorenin concentration and aldosterone (p =0.15), BNP (p =0.17) or eGFR (p =0.16) (Fig. 1). The LVEF was not correlated with the plasma prorenin concentration (p =0.83).

### Plasma prorenin concentration and coronary artery stenosis

**Fig. 2:**
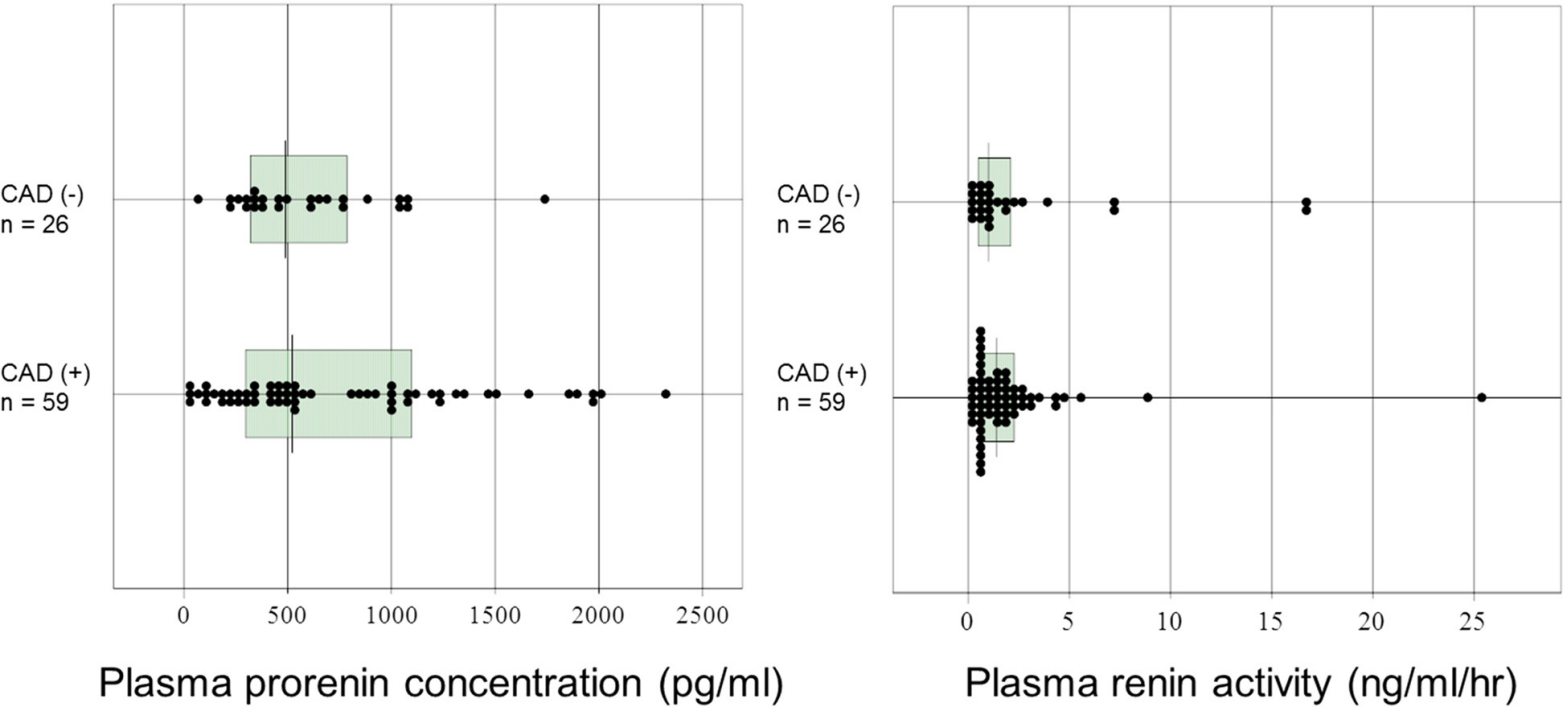
Plasma prorenin concentrations and renin activity in coronary artery disease and non- coronary artery disease groups. Non-parametric data are shown in box plots. A line in each box indicates the median. The upper and lower limits of each box are the 1st and 3rd quartiles, respectively

**Fig. 3:**
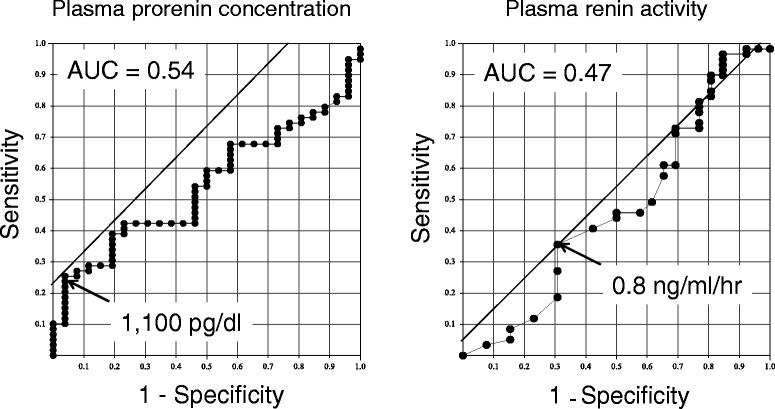
Receiver operating characteristic curves (ROC) analysis. AUC: area under the curve

There was also no significant difference in the plasma renin activity or plasma prorenin concentration between the CAD group and non-CAD group (Fig. 2). There was also no significant difference in the plasma prorenin concentration between patients with and without DM (635 ± 541 and 824 ± 533 pg/ml, respectively). There were also no significant differences in the plasma prorenin concentration in the patients with one-vessel disease, two-vessel disease and three-vessel disease (780 ± 681, 651 ± 507 and 884 ± 580 pg/ml, respectively). However, patients with a high plasma prorenin concentration frequently suffered from CAD (Fig. 2). Receiver-operating-characteristics curve analysis showed that the optimal cutoff value of the plasma prorenin concentration to detect CAD was 1,100 pg/ml with a positive predictive value for estimating CAD of 94 % and a negative predictive value of 36 % (Table 3) (Fig. 3). Only one of 26 patients with elevated plasma prorenin concentration (>1,100 pg/ml) did not have CAD (Fig. 2). The optimal cutoff value of the plasma renin activity to detect CAD was 0.8 ng/ml/hr with a positive predictive value of 72 %, and a negative predictive value of 32 %. A higher plasma prorenin concentration was more accurate than a higher PRA for the detection of CAD. Laboratory and clinical characteristics in the CAD group with patients stratified based on prorenin levels are shown in Table 4.

**Table 3:** Accuracy of plasma prorenin and PRA for detecting coronary artery disease

	Cutoff	Sensitivity	Specificity	PPV	NPV
Prorenin (pg/ml)	1,100	25 (15–34)	96 (92–100)	94 (88–99)	36 (25–46)
PRA (ng/ml/hr)	0.8	36 (26–46)	69 (59–79)	72 (63–81)	32 (22–42)

The optimal cutoff values were determined by receiver-operating-characteristic curve analysis. Data are percentages. Numbers in parentheses are 95 % confidence intervals. PPV: positive predictive value, NPV: negative predictive value. PRA: plasma renin activity

**Table 4:** Laboratory and clinical characteristics in the CAD group with patients stratified based on prorenin levels

	Prorenin ≥ 1,100	Prorenin < 1,100	p-value
	(n = 15)	(n = 44)	
Age, years	72 ± 6	67 ± 9	0.047
Body mass index	24.4 ± 3.3	23.7 ± 2.9	0.43
Prorenin (ng/ml)	1521 (1261–1937)	435 (227–601)	<0.001
Plasma renin concentration (pg/ml)	12.1 (6.9 - 17.5)	5.6 (4.1 - 8.5)	0.001
Plasma renin activity (ng/ml/hr)	2.3 (1.1 - 4.2)	0.8 (0.6 - 1.6)	0.017
Aldosterone (pg/ml)	82 (69–92)	69 (58–104)	0.63
Brain natriuretic peptide (ng/ml)	43 (24–76)	25 (12–60)	0.23
Cystatin C (mg/dl)	1.19 ± 0.54	1.05 ± 0.29	0.21
Creatinine (mg/ml)	0.91 ± 0.34	0.83 ± 0.24	0.32
Estimated GFR (ml/min/1.73 m^2^)	65.8 ± 21.8	70.9 ± 18.3	0.38
Blood urea nitrogen (mg/dl)	17.2 ± 4.5	15.4 ± 3.9	0.16
Hemoglobin A1c (%)	6.2 ± 0.7	6.0 ± 1.2	0.67
Total cholesterol (mg/dl)	178 ± 33	191 ± 33	0.19
LDL cholesterol (mg/dl)	101 ± 29	115 ± 31	0.14
HDL cholesterol (mg/dl)	49 ± 12	48 ± 14	0.74
Triglycerides (mg/dl)	141 ± 60	138 ± 77	0.91
C-reactive protein (mg/dl)	0.13 ± 0.17	0.20 ± 0.22	0.32
Total protein (g/dl)	6.80 ± 0.24	6.70 ± 0.42	0.43
Albumin (g/dl)	4.15 ± 0.24	4.12 ± 0.32	0.74
Sodium (mEq/l)	139.6 ± 2.8	140.6 ± 2.1	0.13
Potassium (mEq/l)	4.27 ± 0.37	3.97 ± 0.32	0.005
Cl (mEq/l)	104.9 ± 3.8	105.5 ± 2.7	0.42

Numerical data are expressed as the mean ± one standard deviation. Non-parametric data are expressed as the median (interquartile range). GFR: glomerular filtration rate

## Discussion

The key findings in the present study were as follows: (1) the optimal cutoff value of plasma prorenin concentration to detect CAD was 1,100 pg/ml with a positive predictive value of 94 % and a negative predictive value of 36 %; (2) patients with high plasma prorenin concentration (>1,100 pg/ml) frequently suffered CAD; (3) the plasma prorenin concentration was positively correlated with the plasma renin concentration; (4) the plasma prorenin concentration was not correlated with LVEF, HbA1c, BNP or eGFR.

### Plasma prorenin concentration and coronary artery disease

Prorenin is an inactive precursor of renin. Both prorenin and renin bind to (pro)renin receptors, and stimulation of these receptors activates intracellular cascades such as MAP kinases ERK1/2 and p38 pathways, and heat shock protein 27. Activation of these pathways leads to enhanced synthesis of DNA, upregulation of TGF-β1, PAI-1, collagen-1, fibronectin and cyclooxygenase-2, independent of the classic renin-angiotensin-aldosterone system [8, 9]. This suggests that stimulation of (pro)renin receptors may exert important effects on the cardiovascular system. (Pro)renin receptors have been localized in various tissues such as the brain, kidneys, heart and vascular smooth muscle cells [10]. Stimulation of (pro)renin receptors has been reported to cause diabetic nephropathy, [3] cardiac injury [4] and vascular damage [5]. Furthermore, the plasma prorenin level was markedly increased in patients with diabetes mellitus with end-organ damage [7, 11]. This suggests that increased plasma prorenin that stimulated (pro)renin receptors resulted in kidney damage.

We measured plasma prorenin concentration in patients with CAD and found that there were patients with CAD that had low plasma prorenin concentration, although patients with high plasma prorenin concentration frequently had CAD. This suggested that there may be two types of coronary stenosis: one type that is sensitive to prorenin and another type that is not. Increased plasma prorenin may have affected coronary artery stenosis through activation of (pro)renin receptors in vascular smooth muscle cells, since activation of (pro)renin receptors has been reported to cause vascular damage [5].

In the present study, patients with a high plasma prorenin concentration (>1,100 pg/ml) frequently suffered CAD (94 %), and accuracy of an elevated plasma prorenin concentration to detect CAD was greater than that of an elevated PRA (>0.8 ng/ml/hr). A previous study demonstrated that high PRA was an independent predictor of major vascular events and mortality in a stable population of high-risk patients with atherosclerosis and/or diabetes [12]. The results of the present study suggested that the plasma prorenin concentration may also be a marker of CAD and/or atherosclerosis. Although, there were patients with CAD that had a low plasma prorenin concentration, patients with a high plasma prorenin concentration frequently had CAD. This may be because a high plasma prorenin concentration induces coronary stenosis by a mechanism that does not involve endothelial injury or lipid accumulation in plaque. A previous study showed that prorenin induced the growth of extracellar matrix components rather than endothelial damage [13]. Another study reported that prorenin enhanced human vascular smooth muscle cell proliferation due to activation of extracellular-signal-related protein kinase in a dose- and time-dependent manner [14]. Therefore, prorenin may induce stenosis from a medial site due to vascular smooth muscle cell proliferation. The detailed mechanisms by which prorenin induces coronary artery stenosis are still unknown. However, the present study provides new insight into the relationship between plasma prorenin and CAD.

### Plasma prorenin concentration and other diseases

Plasma prorenin concentration is determined by the balance between clearance of prorenin from the circulation and production of prorenin. We assessed the relationship between plasma prorenin concentration and eGFR, and there was no correlation between them. This suggests that clearance of prorenin from the kidney is not decreased and a higher plasma prorenin concentration is not due to decreased clearance of prorenin from the circulation. The plasma prorenin concentration was not correlated with LVEF or plasma BNP levels, suggesting that plasma prorenin concentration is not affected by cardiac function.

In the present study, there was also no significant difference in the plasma prorenin concentration between patients with and without DM. However, the plasma prorenin concentration has been reported to be increased in patients who have DM with complications [6]. This discrepancy may be because the present study included DM patients both with and without complications.

### Study limitations

There are several limitations of the present study. First, the relationship between renin and cardiovascular disease may be obscured by the use of various drugs such as ACEIs, ARBs, beta-blockers and diuretics because these drugs modify PRA. In addition, the role of plasma prorenin in patients with CAD is still unclear. Second, a relatively small number of the patients were enrolled in the present study. Thus, the study may have been underpowered to definitively determine the relationship between the incidence of CAD and plasma prorenin concentration. A study in a larger population is needed to elucidate the relationship between the plasma prorenin concentration and the progression of CAD.

## Conclusions

Although, there were patients with CAD that had a low plasma prorenin concentration (≤1,100 pg/ml), patients with a high plasma prorenin concentration (>1,100 pg/ml) frequently had CAD. This may be because a high plasma prorenin concentration (>1,100 pg/ml) induces coronary stenosis by a mechanism that does not involve endothelial injury or lipid accumulation in plaque. The present study is a first report that demonstrated the relationship between plasma prorenin concentration and CAD.

## Abbreviations

PRA: plasma renin activity
CAD: coronary artery disease
HTN: hypertension
DM: diabetes mellitus
LVEF: left ventricular ejection fraction
LVEDd: left ventricular end-diastolic dimension
LVESd: left ventricular end-systolic dimension
BNP: plasma brain natriuretic peptide
HbA1c: hemoglobin A1c
ACEI: angiotensin-converting enzyme inhibitors
ARB: angiotensin II receptor blockers
